# Comparison of the molecular and cellular phenotypes of common mouse syngeneic models with human tumors

**DOI:** 10.1186/s12864-019-6344-3

**Published:** 2020-01-02

**Authors:** Wenyan Zhong, Jeremy S. Myers, Fang Wang, Kai Wang, Justin Lucas, Edward Rosfjord, Judy Lucas, Andrea T. Hooper, Sharon Yang, Lu Anna Lemon, Magali Guffroy, Chad May, Jadwiga R. Bienkowska, Paul A. Rejto

**Affiliations:** 1 Oncology Research & Development, Pfizer Worldwide Research and Development, New York, Pearl River 10965 USA; 2 Oncology Research & Development, Pfizer Worldwide Research and Development, San Diego, CA 92121 USA; 3 Drug Safety Research and Development, Pfizer Worldwide Research and Development, New York, Pearl River 10965 USA

**Keywords:** Syngeneic model, Mutations, Cytolytic activity, Proteomics, Immune infiltration, Neoantigen, IHC, NK cells, Viral proteins

## Abstract

**Background:**

The clinical success of immune checkpoint inhibitors demonstrates that reactivation of the human immune system delivers durable responses for some patients and represents an exciting approach for cancer treatment. An important class of preclinical in vivo models for immuno-oncology is immunocompetent mice bearing mouse syngeneic tumors. To facilitate translation of preclinical studies into human, we characterized the genomic, transcriptomic, and protein expression of a panel of ten commonly used mouse tumor cell lines grown in vitro culture as well as in vivo tumors.

**Results:**

Our studies identified a number of genetic and cellular phenotypic differences that distinguish commonly used mouse syngeneic models in our study from human cancers. Only a fraction of the somatic single nucleotide variants (SNVs) in these common mouse cell lines directly match SNVs in human actionable cancer genes. Some models derived from epithelial tumors have a more mesenchymal phenotype with relatively low T-lymphocyte infiltration compared to the corresponding human cancers. CT26, a colon tumor model, had the highest immunogenicity and was the model most responsive to CTLA4 inhibitor treatment, by contrast to the relatively low immunogenicity and response rate to checkpoint inhibitor therapies in human colon cancers.

**Conclusions:**

The relative immunogenicity of these ten syngeneic tumors does not resemble typical human tumors derived from the same tissue of origin. By characterizing the mouse syngeneic models and comparing with their human tumor counterparts, this study contributes to a framework that may help investigators select the model most relevant to study a particular immune-oncology mechanism, and may rationalize some of the challenges associated with translating preclinical findings to clinical studies.

## Background

Preclinical mouse models support cancer therapeutic development by contributing to target validation, elucidation of drug mechanism of action, and generation of biomarker hypotheses to test in clinical settings. Two major categories of preclinical mouse models are immune compromised and immune competent [1]. Patient-derived xenografts (PDXs) and cell-line derived xenografts (CDXs) arise by transplanting either human tumor explants or established human tumor cell lines into immune deficient mouse hosts, and have been widely applied in developing cancer therapies that modulate tumor cell autonomous functions. The rich genetic information about cancer cell lines [2] and PDXs [3, 4] from extensive genomic characterization supports model selection to investigate specific target biology or perform drug sensitivity screens. CDXs and PDXs have limited use in cancer immunotherapy studies because an immune compromised host is required for xenotransplantation. By contrast, immune competent mouse model systems such as syngeneic mouse models, derived by transplanting established mouse cell lines or tumor tissues to strain-matched mouse hosts, and genetically engineered mouse models (GEMMs), created by introducing genetic modifications that result in spontaneous tumor development, retain intact mouse immune systems and are better suited to study the interplay between immune and tumor cells.

The recent approval of immune checkpoint inhibitors and their success in generating durable response in some patients has reinvigorated interest in developing novel immune therapies and evaluating combination regimens [5]. While syngeneic mouse models and GEMMs both possess intact immune systems [6], GEMMs typically have relatively few mutations and lower immunogenicity. Syngeneic mouse models have a broader spectrum of mutations and have served as workhorses for investigating immune therapies and studying the intricate immune surveillance of cancer development [6, 7]. Anti-tumor activity via checkpoint blockade, such as with a CTLA4 blocking antibody, was initially observed in syngeneic models [8], suggesting that syngeneic model findings may translate to the clinic. The anti-CTLA4 antibody has variable response among different syngeneic models with marked response in CT26, GL261, and EMT6, while it was shown to be ineffective in B16F10, a melanoma model [8]. This response pattern was postulated to be due to the diverse immunogenicity of the models, although the underlying molecular mechanisms remain elusive due in part to a lack of understanding of the immunogenic state that favors response. Compared to patient-derived xenograft models, there have been far fewer syngeneic models established and characterized, although recently several studies have been published that begin to profile the molecular and cellular characteristics of immune competent mouse models [9–13].

We compared genomic, proteomic and immunohistochemistry (IHC) features of a panel of ten commonly used mouse syngeneic models [6, 10, 13] with the corresponding features of human tumors in The Cancer Genome Atlas (http://cancergenome.nih.gov/). We characterized the mutational landscape and predicted the neoantigen burden of these models through whole exome sequencing, and compared the variants identified in syngeneic models to common driver mutations in human tumors. We evaluated syngeneic model tumor phenotypes through immunohistochemistry and compared the architecture to human cancers, performed RNA-Seq of tumors grown in syngeneic mice as well as the same cells grown in culture, and predicted immune infiltration through computational deconvolution of gene expression data into immune components. Compared to previous studies [9–13], our study includes expression analysis for syngeneic models from both cells grown in vitro culture as well as in vivo tumor samples, enabling an assessment of tumor cell intrinsic properties. We also characterized these mouse syngeneic models by proteomics which enabled us to verify gene expression findings identified from transcription profiling, as well as to identify potential mouse virus proteins that may contribute to immunogenicity.

## Results

### Commonly used mouse syngeneic models in this study do not fully recapitulate common driver mutations in human tumors

We performed whole exome sequencing (WES) of ten syngeneic models commonly used in immune oncology preclinical studies (Table 1, Additional file 1: Table S1) and characterized the missense mutations (Additional file 2: Figure S1). To assess the accuracy of our variant calls, we tested 115 variants mapped to the TARGET (tumor alterations relevant for genomics-driven therapy) database (http://archive.broadinstitute.org/cancer/cga/target) by Sanger sequencing. All 115 of the predicted variants were validated (Additional file 3: Table S2), supporting a high level of precision for our variant calls. The transition/transversion (Ts/Tv) ratio varied across a wide span ranging from 0.27 to 3.65 (Additional file 2: Figure S2A), similar to the Ts/Tv range in somatic variants from human cancers (Additional file 2: Figure S2A) [14]. For example, MC38 has many more transversions than transitions, while more than 50% of the SNVs for the CT26 model are C > T;G > A transitions (Additional file 2: Figure S2B). The broad Ts/Tv range in syngeneic models may reflect the variety of mutagens used to derive these models. MC38 was induced by the DNA methylating agent DMH and enriched with C > A;G > T transversions while the CT26 model was generated by the carcinogen NMU, known to induce C > T;G > A mutations [15]. Previously, both transversion and transition mutations were reported to be induced by DMH in mouse Trp53 genes [16].

**Table 1:** Mutational load of the 10 syngeneic mouse models and the corresponding human cancer (LUAD: Lung adenocarcinoma, LUSC: Lung squamous cell carcinoma)

Model	Tumor Type	Parent Strain	Origin	Mutational Load (per Mb)	Median Human Mutational Load (per Mb)
4T1	breast	BALB/c	virus	5	1
A20	B-cell lymphoma	BALB/c	spontaneous	20	NR
CT26	colorectal	BALB/c	carcinogen	56	5
RENCA	renal	BALB/c	spontaneous	46	1.6
EMT6	breast	BALB/c	virus	13	1
EL4	T-cell lymphoma	C57BL/6	carcinogen	51	NR
MC38	colorectal	C57BL/6	carcinogen	75	5
LLCsr	lung	C57BL/6	spontaneous	72	5.3 (LUAD) 7.5 (LUSC)
B16F10	melanoma	C57BL/6	spontaneous	35	9.6
F9	teratocarcinoma	129S6/SvEv	embryoimplantation	11	NR

NR: not reported because there is no direct human equivalent data

**Fig. 1:**
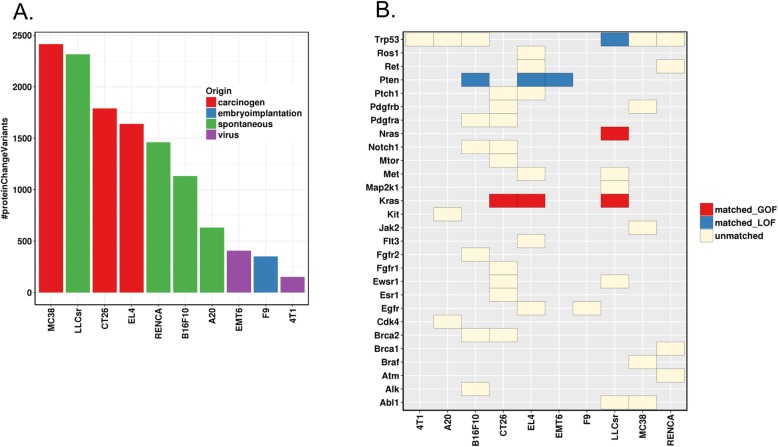
**a** Variants predicted to alter protein functions (variant effect defined as MODERATE, “A non-disruptive variant that might change protein effectiveness”, or HIGH, “The variant is assumed to have disruptive impact in the protein, probably causing protein truncation, loss of function or triggering nonsense mediated decay”, by SnpEff). **b** Protein sequence altering variants of known cancer genes; GOF: gain of function; LOF: loss of function; matched_GOF: mouse variants matching human GOF variants (exact variants); matched_LOF: mouse variants matching human LOF variants (truncating mutation or missense mutation at the same amino acid); unmatched: mouse variants not reported as known actionable variants in human tumors

Mutational load has been correlated with tumor immune infiltrates [17] and clinical response of checkpoint blockades in some human tumors [18, 19]. We classified mutations into four categories using snpEff [20] based on their predicted impact on protein functions: high (“The variant is assumed to have disruptive impact in the protein, probably causing protein truncation, loss of function or triggering nonsense mediated decay”), moderate (“A non-disruptive variant that might change protein effectiveness”), low (“Assumed to be mostly harmless or unlikely to change protein behavior”), or modifier (“Usually non-coding variants or variants affecting non-coding genes, where predictions are difficult or there is no evidence of impact”). Next, we calculated mutational load for the “high” and “moderate” mutations (Fig. 1a), and compared with the nonsynonymous mutational load of the corresponding human tumors. In general, the mutational load for “high” and “moderate” mutations in syngeneic models was higher than the median nonsynonymous mutational load in human tumors, although the values are within the range in human tumors (Table 1). MC38 has the highest mutational load, followed by LLCsr and CT26, while EMT6, F9 and 4T1 have the lowest mutational load. As expected, the carcinogen-induced models tend to have the highest mutational burden, followed by spontaneously generated tumors, with viral induced models bearing the lowest mutational load (Fig. 1a).

We focused on genes that lead to carcinogenesis when altered in human tumors and compared 43 point mutations across 27 genes whose human orthologs are reported in the TARGET database and annotated as actionable in OncoKB (http://oncokb.org/), as well as 8 variants of the tumor suppressor Trp53. Only four point mutations in two oncogenes (Kras G12C:LLCsr, G12D:CT26, G13D:EL4, and Nras Q61H:LLCsr) and four point mutations in two tumor suppressor genes (Pten T131P:B16F10, R130W:EL4, G209*:EMT6, and Trp53 E32*:LLCsr) in the mouse syngeneic models had corresponding mutations (the exact variant in Kras and Nras, a truncating mutation or missense mutations at the same amino acid in Pten and Trp53) in human tumors regardless of the tissue of origin (Fig. 1b). Next, we investigated whether genes frequently mutated in human tumors are also mutated in syngeneic models of the same tissue of origin. While KRAS, APC and TP53 are frequently mutated in human colon tumors, CT26 had homozygous Kras mutations (G12D, V8M) and did not have mutations in Apc or Trp53; MC38 had Trp53 heterozygous mutations (G242 V, S258I) and a Smad4 heterozygous mutation (G351R), mutated in approximately 12% of human colon cancer, with no mutations in Kras or Apc (Table 2). Neither colon syngeneic model has an APC mutation, which is mutated in the majority of human colorectal cancer and neither the breast-derived tumor model EMT6 nor 4T1 have activating mutations in PIK3CA. EMT6 and 4T1 contain fewer protein altering mutations than other syngeneic models, although 4T1 has an insertion in Trp53 that results in a frameshift mutation (E32fs). The LLCsr model also contains mutations in Trp53 (E32*, R334P) as well as Kras (G12C). Unlike CT26, the Kras (G12C) mutation in LLCsr is a heterozygous mutation. By contrast, the V600 BRAF mutation, a mutation common in human melanoma, was not identified in the melanoma B16F10 model. Similarly, genes frequently mutated in human kidney cancer such as VHL were not identified in the RENCA model.

**Table 2:** Frequently mutated human cancer genes and their mutations in syngeneic models of the same cancer type

Human Cancer	Human Gene (^a^)	Human Mutation Frequency	Model	Model Mutation	Model	Model Mutation
BRCA	PIK3CA	32.48	4T1	NA	EMT6	NA
BRCA	TP53	30.65	4T1	p.Glu32fs	EMT6	NA
BRCA	CDH1	11.41	4T1	NA	EMT6	NA
COAD	APC	71.62	CT26	NA	MC38	NA
COAD	TP53	53.6	CT26	NA	MC38	p.Gly242Val p.Ser258Ile
COAD	KRAS	43.24	CT26	p.Gly12Asp p.Val8Met	MC38	NA
COAD	FBXW7	17.12	CT26	NA	MC38	NA
COAD	PIK3CA	14.86	CT26	NA	MC38	NA
COAD	SMAD4	11.71	CT26	NA	MC38	p.Gly351Arg
COAD	ATM	11.26	CT26	NA	MC38	NA
SKCM	BRAF	51.23	B16F10	NA		
SKCM	NRAS	26.7	B16F10	NA		
SKCM	ROS1	17.98	B16F10	NA		
SKCM	ERBB4	16.35	B16F10	NA		
SKCM	TP53	15.26	B16F10	p.Asn128Asp		
SKCM	KDR	13.35	B16F10	NA		
SKCM	NF1	12.81	B16F10	NA		
SKCM	CDKN2A	12.26	B16F10	NA		
LUSC	TP53	81.46	LLCsr	p.Glu32* p.Arg334Pro		
LUSC	PIK3CA	15.17	LLCsr	NA		
LUSC	CDKN2A	14.04	LLCsr	NA		
LUSC	NF1	11.8	LLCsr	NA		
LUSC	ROS1	10.67	LLCsr	NA		
KIRC	VHL	49.89	RENCA	NA		

^a^Genes from TARGET database with at least 10% mutation frequency in TCGA samples

*BRCA* Breast invasive carcinoma, *COAD* Colon adenocarcinoma, *SKCM* Skin cutaneous Melanoma, *LUSC* Lung squamous cell carcinoma, *KIRC* Kidney renal clear cell carcinoma

### Some syngeneic tumors display a mesenchymal-like phenotype

**Fig. 2:**
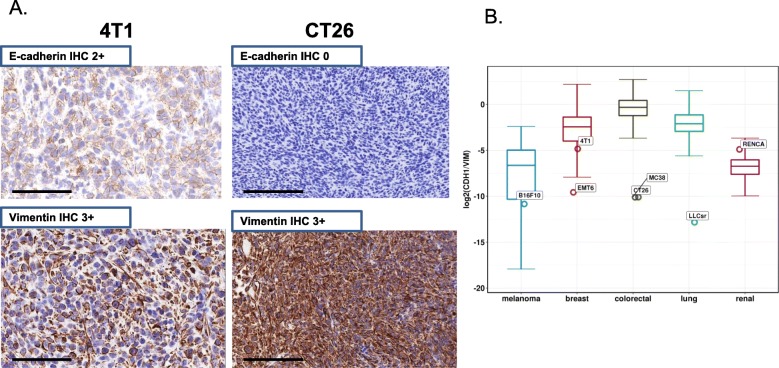
Mesenchymal-like phenotype of some syngeneic tumors. **a** E-cadherin and vimentin stain in 4T1 and CT26 model. **b** Comparison of ratio of E-cadherin vs vimentin between solid tumor syngeneic models (open circle) with tissue matched human tumors from TCGA (boxplot; lung: lung adenocarcinoma and lung squamous cell carcinoma). Ratio was calculated with the expression value (TPM) of E-cadherin and vimentin

In addition to genetic features, we compared the tumor histology of these mouse syngeneic models with human tumors. The in vivo tumors were stained with E-cadherin antibodies, an epithelial cell marker, and vimentin, a marker for cells undergoing epithelial to mesenchymal transition. Many models had high vimentin expression suggesting a more mesenchymal-like phenotype (Fig. 2a, Additional file 2: Figure S3). In addition, the ratio of E-cadherin to vimentin is much lower than the corresponding human tumors in TCGA with the exception of RENCA (Fig. 2b), suggesting that syngeneic models typically have a more mesenchymal-like tumor cellular phenotype than human tumors.

### These syngeneic models have relatively low T-lymphocyte infiltration

**Fig. 3:**
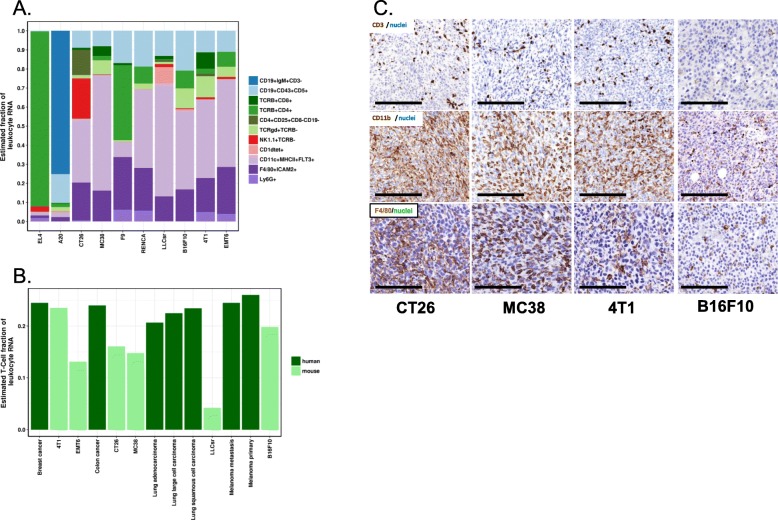
Immune subsets in syngeneic models. **a** In silico immune cell deconvolution of syngeneic tumor samples. Syngeneic models exhibited various immune cell type infiltrations with major NK cell infiltration predicted in CT26 models. **b** Comparison of estimated total T-cell fraction of leukocyte in selected mouse syngeneic models and their corresponding human tumors. Human data were downloaded from Gentles et al. [22]. Total T-cell fraction plotted here is the sum of all predicted T-cell subsets including CD4+, CD8+, Treg, and gamma-delta T-cells. **c** CD3 staining for T-cells, CD11b staining for myeloid cells, and F4/80 staining for macrophage

The baseline immune infiltration of a panel of syngeneic models (Table 1) was evaluated by transcription profiling and chromogenic IHC. We performed RNA-Seq for syngeneic tumors grown in vitro culture and in vivo (Additional file 4: Table S3), and implemented an in silico immune cell deconvolution using a nu-support vector regression (nuSVR) developed for mouse samples that is similar to approaches recently developed for human samples [21]. As expected, a large percentage of T cells and B cells were predicted for EL4 and A20, T cell and B cell lymphoma models, respectively. A relatively high percentage of myeloid infiltration along with a relatively low percentage of T cells was predicted by in silico immune cell deconvolution (Fig. 3a). The T-cell fraction was lower in most syngeneic models compared to the corresponding human tumors [22] (Fig. 3b). Furthermore, there were high levels of myeloid and macrophage infiltration by IHC in these models (anti-CD11b or anti-F4/80 staining, Fig. 3c).

### Predicted neoantigen load in these syngeneic mouse models does not correlate with cytolytic activity

Neoantigen load has been reported to correlate with tumor immune infiltrates [17] and clinical response of checkpoint blockades in some human tumors [18, 19]. We developed a neoantigen prediction pipeline based on MHC class I binding for the syngeneic models (details in method section); the number of predicted neoantigens correlates with mutational load (Additional file 2: Figure S4A) as in human tumors. Next, we evaluated the relationship between the predicted neoantigen load and tumor immunity using the cytolytic activity (CYT) as an indicator of the tumor immunity. We defined the cytolytic activity to be the log average (geometric mean) of two key cytolytic effectors, granzyme A (GZMA) and perforin (PRF1) [17]. Unlike what has been reported for human tumors, we did not observe a significant correlation between the neoantigen load and cytolytic activity (Additional file 2: Figure S4B).

### Relative immunogenicity of syngeneic tumors in our study differs from their tissue of origin in human tumors

**Fig. 4:**
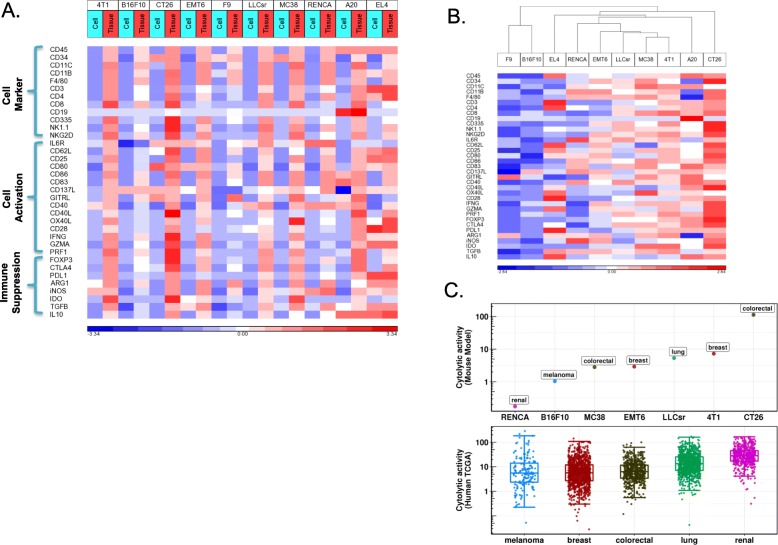
Immune infiltration in syngeneic models. **a** Gene expression of immune cell type, immune cell activation and immune suppression markers in cells grown in vitro and tumor tissues from the transplantation. Gene expression shown as log2 of transcript per million (TPM) and standardized across samples. **b** Unsupervised clustering analysis of immune marker expression in tumor tissues from the transplantation separates syngeneic models into high and low infiltration models. **c** Comparison of cytolytic activity of solid tumor syngeneic models with tissue matched human tumors from TCGA (human data were downloaded from Rooney et al. [17]).Cytolytic activity (CYT) is defined as the log-average (geometric mean) of Gzma and Prf1 expression in transcripts per million (TPM) as describe by Rooney et al.

We investigated the relative immunogenicity among syngeneic tumors using RNA-Seq and proteomics. Gene expression of many markers of immune cells, immune activation and suppression were dramatically up-regulated in tumors in vivo compared to the corresponding cells in vitro, consistent with immune infiltration (Fig. 4a). Unsupervised hierarchical clustering of these immune-related genes displayed differential immune infiltration among models (Fig. 4b) where CT26, a colon cancer model, and 4T1, a breast cancer model, had the highest immune infiltration compared to other models while B16F10, a melanoma model and F9, a testicular teratoma, had lower immune infiltration. Total leukocyte infiltration in syngeneic models by CD45 (PTPRC) expression from RNA-Seq had a similar trend as did cytolytic activity, another indicator of cancer immunity, which was also highest in CT26 and 4T1 and lowest in B16F10 and RENCA among the solid tumor models (Fig. 4c). CT26 was highly responsive to CTLA4 checkpoint inhibitors, but not to PD-1 inhibitors, while other models including the B16F10 melanoma model did not respond significantly to either of the checkpoint inhibitors (Additional file 2: Figure S5). The high immunogenicity of the CT26 model and low immunogenicity of B16F10 and RENCA models in our study differs from what has been reported in human tumors from the corresponding tissue of origin, where kidney cancer has the highest median cytolytic activity. Although human colon tumors and melanoma have similar median cytolytic activity, melanoma has a much more skewed distribution where a significant fraction of tumors have high cytolytic activity (Fig. 4c). CT26 had dramatically higher expression of Gzma (Additional file 2: Figure S6A), and also the highest cytolytic activity as well as Gzma expression based on our proteomic analysis (Additional file 2: Fig. S6B, C). The CT26 model was predicted to have significant NK cell infiltration based on in silico immune cell deconvolution of RNA-Seq (Fig. 4a) which is consistent with high Gzma expression and corresponding high cytolytic activity, as Gzma has been previously shown to be expressed prominently in NK cells in mouse (http://www.immgen.org/).

**Fig. 5:**
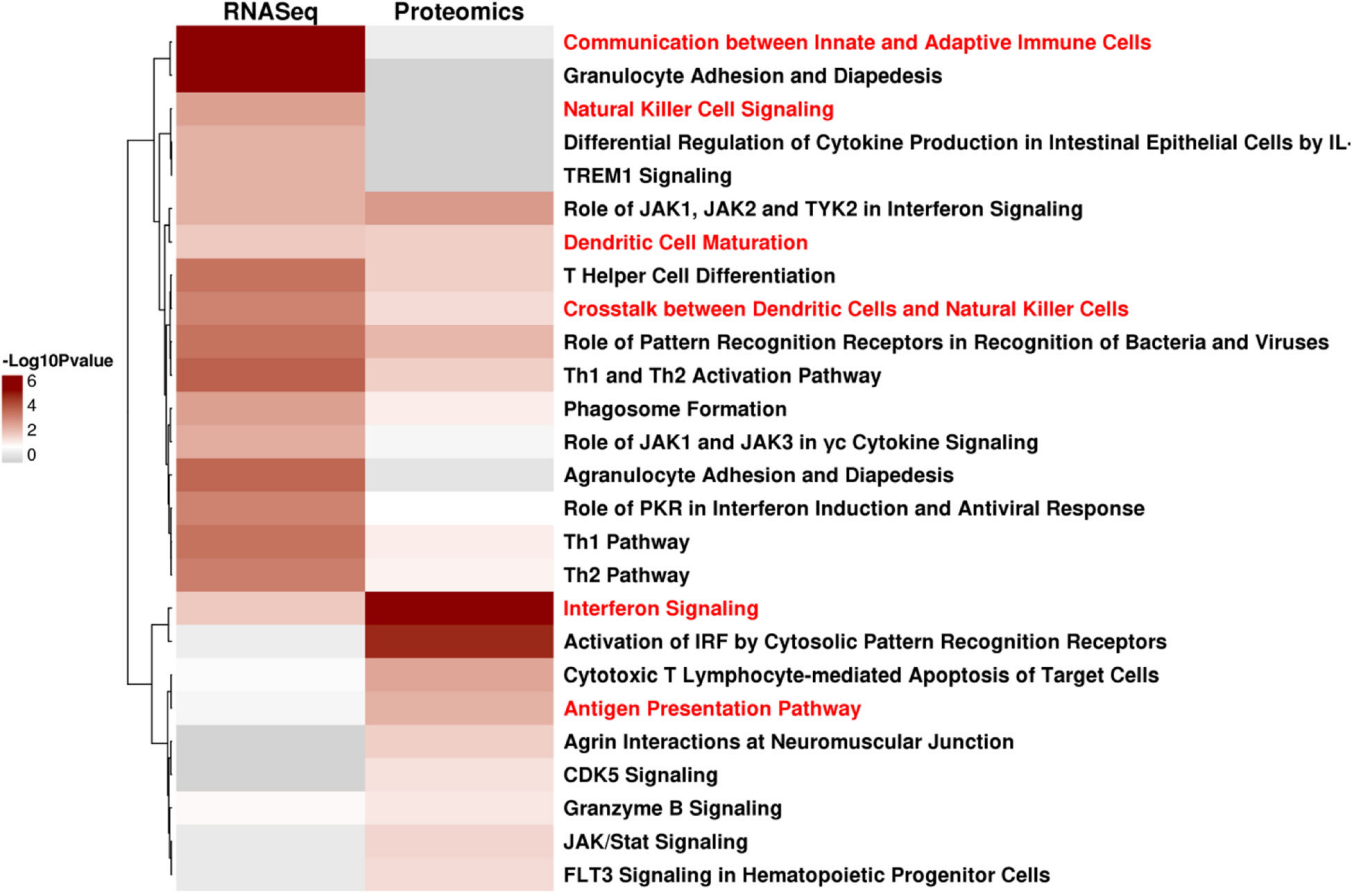
Top 10 significantly enriched pathways of genes up-regulated in CT26 in vivo tumor samples compared to in vivo tumor samples of other syngeneic models and CT26 in vitro samples from either RNA-Seq or proteomics data analysis (Fisher Exact *p*-value <= 0.05)

To further investigate the unique biology of the CT26 model, we analyzed pathways enriched in genes up-regulated in CT26 tumor samples in vivo compared to the same CT26 cells grown in vitro culture and other syngeneic in vivo tumors utilizing RNA-Seq. We identified NK-related pathways including “Crosstalk between Dendritic Cells and Natural Killer Cells” and “Natural Killer Cell Signaling” to be significantly enriched (Fig. 5). Further analysis identified the “Crosstalk between dendritic cells and Natural Killer Cells”, “Interferon signaling”, and “Dendritic cell maturation” pathways as enriched both from RNA-Seq and proteomics. Our integrated pathway analysis is consistent with increased natural killer cell signaling in the CT26 model (Fig. 5). Contrary to the large NK cell infiltration in the CT26 colon model, NK cell infiltration has been reported to be much lower (approximately 1–3%) in human colon tumors [22].

### Proteomics characterization of virus antigen

**Fig. 6:**
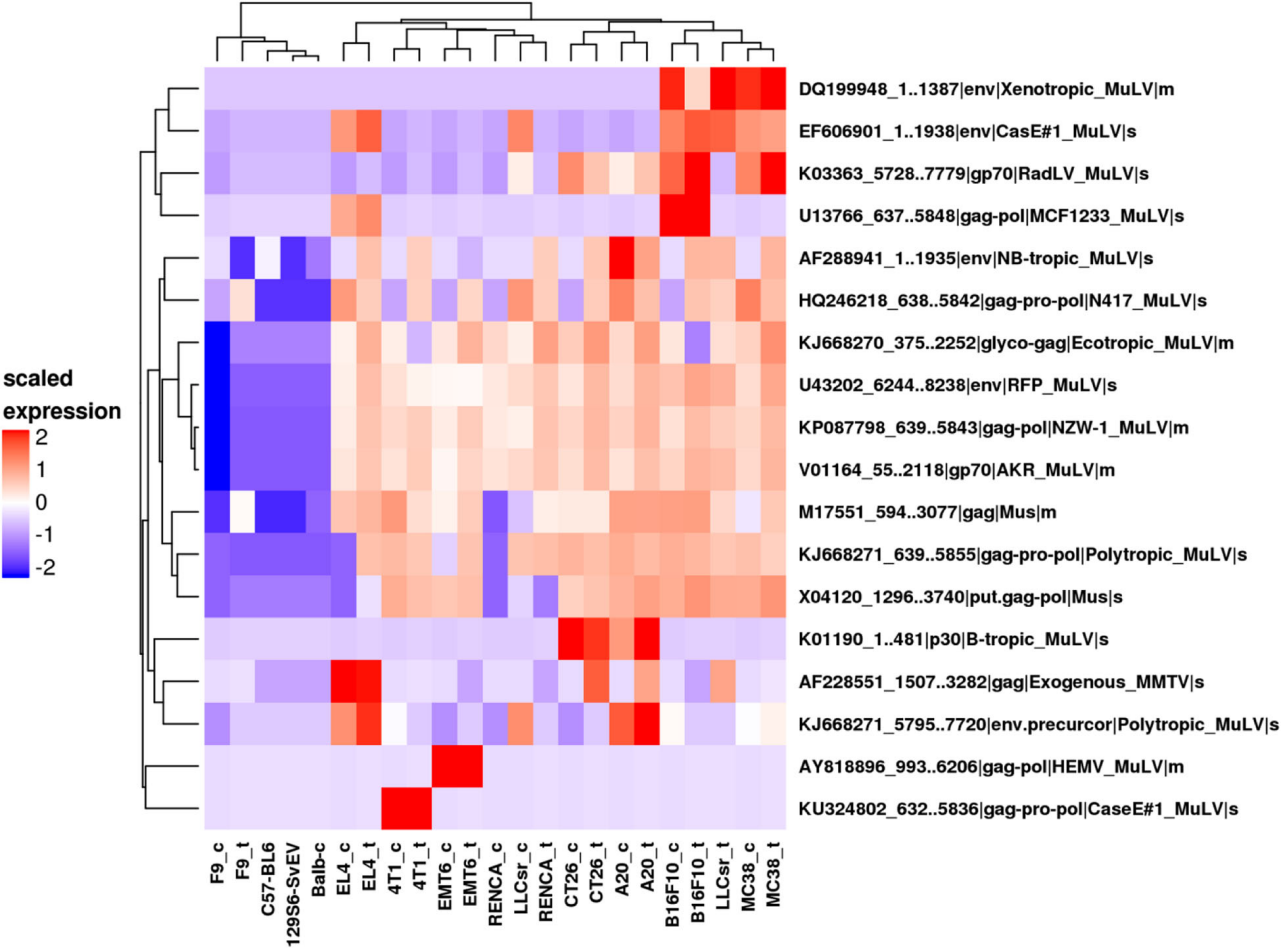
Viral peptides in syngeneic in vitro and in vivo samples from proteomic analysis (s: virus protein detected in soluble fraction; m: virus protein detected in membrane fraction, _t: in vivo tumor sample, _c: in vitro sample. Non-tumor samples are from the tails of the parent strain). Hierarchical clustering using euclidean distance and complete linkage clustering method of log2 transformed and scaled LFQ values

Since viral antigens may also contribute to immunogenicity, we evaluated mouse viral proteins using custom proteomics. With the exception of LLCsr where a gag protein of mouse mammary tumor virus (ENA|AF228551_1507..3282) was detected in the tumor in vivo but not in vitro (Fig. 6), 18 mouse virus proteins were detected in cell lines with similar expression patterns when grown either in vitro or in vivo. Sixteen virus proteins were recurrent in more than one model while two (AY818896_993..6206, KU324802_632..5836) were expressed in only a single model. One of the viral proteins that is broadly expressed in 9 out of 10 models, murine leukemia virus envelope gp70 (ENA|V01164_55..2118), has been previously reported to be broadly expressed in mouse cancer cells (Scrimieri et al. 2013). F9, a testicular teratoma, had very little virus protein expression compared to other models.

## Discussion

Syngeneic models are widely employed for evaluating efficacy, exploring mechanism of action, and generating predictive biomarker hypotheses to inform clinical development. We investigated the molecular and cellular properties of ten commonly used syngeneic models in immune oncology preclinical studies. While these models possess molecular features similar to human cancers as assessed by next generation sequencing, proteomics and immunohistochemistry, they also have a number of distinct properties: 1) only a small fraction of the somatic SNVs from the commonly used mouse syngeneic models that we studied have the specific actionable mutation found in human tumors, 2) syngeneic models from our studies have lower T-lymphocyte infiltration compared to their corresponding human cancers, and 3) these syngeneic models have a more mesenchymal-like phenotype than human tumors. Commonly used mouse syngeneic models derived from a particular tissue typically do not reflect the characteristics of major human tumor populations in the corresponding tissue. For example, the mouse colon tumor model CT26 has the highest immunogenicity and is the most responsive model to CTLA4 treatment among the models tested while the melanoma model B16F10 had the lowest immunogenicity and no response to the checkpoint inhibitors tested in our study, in contrast to the relatively high level of immunogenicity and response to checkpoint inhibitors for human melanomas. These differences suggest that these commonly used mouse syngeneic models derived from a particular tissue of origin do not fully recapitulate features of human tumor populations from the same tissue, and translation from mouse to human based on preclinical studies may be more subtle than simply selecting a model from the same tissue of origin.

Many common mutations in human tumors are not represented in the ten mouse syngeneic models that we studied (Table 2). Although some known human cancer genes are mutated in these mouse syngeneic models, they rarely contain the same actionable variant observed in human tumors (Fig. 1b). For example, of the three frequently mutated genes in human colon tumors, KRAS, APC and TP53, the CT26 colon tumor model only had activating Kras mutations (G12D, V8M), and the MC38 colon tumor model only had Trp53 mutations (G242 V, S258I). While more than 70% of human colon tumors have mutations in APC, a common early event in the evolution of human colon cancer [23], neither of the two colon syngeneic mouse models that we studied contain APC mutations (Table 2). The V600E BRAF mutation, a common oncogenic mutation in human melanoma, was not detected in the B16F10 melanoma model. These findings suggest that some of these common syngeneic models may not fully recapitulate the genetic origin of major population of human cancers, and these limitations may present challenges for modeling combination therapies of immune-oncology and targeted agents. The lack of genetic representation of major human populations in these models could be due to the limited number of models studied, the highly heterogenous human tumor population, as well as the mechanism by which these models were derived. For example, B16F10 is derived from spontaneous melanocytic tumors, while human melanoma frequently arises from UV-exposure. Although the B16F10 model lacks mutations in BRAF, NRAS, and NF1, it may be useful to study TP53 mutant or triple wild type human melanoma [24]. The high mutational load of CT26 and MC38 models may represent specific hypermutated human colon tumors. Overall, these models could represent human tumor subpopulations and be used to study them preclinically.

We analyzed the mutational and neoantigen load of these ten common syngeneic models by whole exome sequencing. While syngeneic models tend to bear a higher mutational load than the median mutational load in human tumors in TCGA, they are still within the observed range (Table 1). Our analysis shows that mutational load is highly correlated with neoantigen load, suggesting that mutational load can be a surrogate for neoantigen load. However, unlike what has been reported in human, we did not observe a significant correlation of neoantigen load with immunogenicity or response to checkpoint blockade. This difference could be due to the difference in the activity of mouse surrogate antibodies relative to antibodies used in human studies, the limited number of models tested, or the diverse origin and tumor types represented in this study.

We also observed cellular phenotypic differences between syngeneic mouse models and human tumors. These models had a more mesenchymal than epithelial phenotype compared to human tumors, and in silico deconvolution showed various immune infiltration patterns among these 10 models. The diversity of immune infiltration makes it possible to test the effect of therapies targeting different immune cells on the tumor growth, although these models differ from human tumors with relatively low T-lymphocyte infiltration and high myeloid infiltration in several of the syngeneic models analyzed in this study, along with a lower T-cell fraction than the corresponding human tumors. One potential explanation of the lower T cell infiltration observed in syngeneic models could be the implantation method used in our study. With the exception of 4T1 and EMT6, which were implanted via mammary fat pad, the remainder were implanted subcutaneously. While subcutaneous implantation is widely used in preclinical immune-oncology studies, orthotopic implantation may create a tumor microenvironment more comparable to human cancers. Since these syngeneic mouse models have relatively low T-lymphocyte infiltration, they may represent T-cell excluded human tumors.

In addition to neoantigens from somatic mutations, neoantigens from endogenous viral proteins can also act as tumor associated antigens and elicit CD8 T cell immunity. We identified viral proteins from two different types of oncogenic viruses that are uniquely expressed in tumor models but not in skin epithelia from the tails of normal mice: murine leukemia virus and murine mammary tumor virus (Fig. 6). Two of the viral proteins were selectively expressed in specific models, suggesting differences in pathogen exposure for each model when it was originally derived. While these mouse specific viral antigens may play a role in cancer immunity in these models, the specific immunity of these viral antigens remains to be investigated.

Our study suggested that the relative immunogenicity of various tumor types among syngeneic models we studied differs from human tumors: the colon model CT26 is the most immunogenic while the melanoma model B16F10 is the least immunogenic. Recent studies [25, 26] implicated activation of the β-catenin oncogenic pathway as inducing resistance to anti-tumor immunity in melanoma. Consistent with this mechanism, the B16F10 model had high β-catenin expression while highly immunogenic models such as CT26 had lower expression (Additional file 2: Figure S7). Epigenetic silencing has also been reported to limit T cell immunity in ovarian and colon cancer [25, 27, 28]. Ezh2, a member of the polycomb repressive complex 2 (PRC2) and Dnmt1, a DNA methylation enzyme were most highly expressed in the B16F10 model (Additional file 2: Figure S7), implicating both β-catenin pathway activation and epigenetic silencing as potential tumor intrinsic mechanisms leading to resistance to checkpoint inhibitors in this model. The B16F10 model may represent melanoma patients who do not respond to checkpoint blockade, and may serve as a model for evaluating combination therapies of checkpoint inhibitors with agents that target these reported tumor intrinsic resistance mechanisms.

Unlike most human colon tumors, the CT26 colon tumor model had the highest immunogenicity among the models evaluated in our study. CT26 has high expression of immune markers and elevated cytolytic activity compared to other syngeneic models, consistent with previous reports of CT26 as a highly immunogenic model [13]. The high cytolytic activity in CT26 is largely attributed to high Gzma expression. In contrast to human, where Gzma can be expressed in both NK cells and CD8+ T cells, Gzma is predominantly expressed on NK cells in mouse (http://www.immgen.org/). Concordantly, our in silico immune cell type deconvolution identified significant NK cell infiltration in the CT26 model. Moreover, our integrated pathway analysis of both mRNA and protein expression identified several pathways related to NK cell function as highly enriched in CT26 tumors compared to either CT26 cells in vitro or other syngeneic tumors, providing further evidence of NK cell infiltration in the CT26 model that may contribute to its cytolytic activity. Antigen presentation and dendritic cell function are more active in CT26 compared to other models (Fig. 5), and CD80 was expressed on CT26 cancer cells (Fig. 4a). Besides its well-known function as a costimulatory molecule for T cell activation, CD80 has been reported to play a role in NK cell activation in both human and mouse cell lines expressing CD80 [29, 30]. In addition, CD28 and CTLA4 expression has been reported in activated mouse NK cells, the interaction between CTLA4 and CD80 has a direct effect on IFN-γ release by NK cells, and CTLA4 expression has been reported in mouse tumor infiltrating NK cells [31]. Furthermore, we observed significant CTLA4 expression in CT26 in vivo tumor samples (Additional file 2: Figure S8) and on some tumor infiltrating NK cells based on single-cell RNA-Seq (data not shown). The role of NK cells in the remarkable response of CT26 to CTLA4 blockade (Additional file 2: Figure S5) as well as the potential mechanism of NK activation through CD80 expressed on CT26 cancer cells remains to be elucidated by future experiments.

## Conclusions

We profiled the gene expression, proteomic, cellular phenotype, and pharmacology of several checkpoint inhibitors in ten commonly used syngeneic models. We found both similarities as well as important differences between commonly used syngeneic models and the corresponding human tumor from the same tissue of origin. While these syngeneic models do not fully recapitulate the biology of human tumors, they may mimic specific human cancer segments. The differences between these syngeneic models commonly used in immune oncology preclinical studies and human cancer may require interpretation to translate preclinical findings from these models to the clinic beyond simply matching the tumors from the same tissue of origin.

The limitations of common syngeneic models present opportunities for further development to establish additional immune competent mouse models that harbor oncogenic driver mutations and encompass mutational loads more representative of human tumors. While GEMM models are typically generated through genetically engineering of driver gene mutations, they often do not recapitulate the mutational burdens of human cancers and in most cases are less immunogenic. One approach to increase the mutational burden of the GEMM models is through CRISPR knockout of genes in the DNA mismatch repair pathway. Investigators have reported enhanced T cell infiltration of Msh2^KO^ tumors at early time points in Msh2 knockout GEMM models that also have Kras and Trp53 mutations (KP) [32]. Alternatively, a melanoma model YUMMER1.7 has been derived through irradiation of the genetically engineered mouse melanoma YUMM1.7 cell line, which harbors three driver mutations: Braf^V600E^, Pten^−/−^ and Cdkn2a^−/−^, and has been reported to have increased T cell infiltration and response to immune check point inhibition [33] . While syngeneic models provide an opportunity to evaluate fundamental immunological pathways in the context of malignancy and have an important role in the study of novel therapeutics, they should be applied carefully with consideration of their differences from human tumors when informing clinical strategies.

## Methods

### Animals

Female inbred BALB/cAnNCrl 6–10 weeks of age were purchased from Charles River Laboratories (strain code 028). Female inbred C57BL/6 J mice 6–10 weeks of age were purchased from Jackson Labs (strain 664). Female inbred 129S6/SvEvTac mice 6–10 weeks of age were purchased from Taconic Laboratories. All mouse strains were housed under specific pathogen-free conditions in Tecniplast IVC Green Line IVC cages in the vivarium of a Pfizer location in Pearl River, New York. Mice were housed on a 12:12 light:dark cycle, with ad libitum UV-sterilized water and low isoflavone 5 V02 IF 50 irradiated Purina Chow (Purina). Animals were monitored twice daily for health status. No adverse events were observed. At the start of the experiments mice weighed 18 – 25 grams. All animal studies were approved by the Pfizer Institutional Animal Care and Use Committee (IACUC) in accordance with the guidelines described in “Guide for the Care and Use of Laboratory Animals” (NRC, 2011).

### Syngeneic mouse models

4T1, A20, CT26, RENCA, EMT6, B16F10, and F9 cells were obtained from the American Type Culture Collection, Manassas, Virginia. MC38 cells were obtained from the laboratory of Antoni Ribas’ laboratory at UCLA, Los Angeles, California. LLCsr (Lewis lung carcinoma) cells were obtained from the laboratory of Shahin Rafii, Department of Genetic Medicine, Ansary Stem Cell Institute, Weill Cornell Medical College, New York, NY. For all models except the breast cancer model 4T1 and the colorectal model CT26, cells were injected in a 200 μl cell suspension in PBS in the right flank of 7–10 week old female mice. To establish the colorectal syngeneic model MC38, 1 × 10^6^ cells were implanted into C57BL/6 J mice. To establish the lung cancer model LLCsr, the melanoma model B16F10, or the T cell lymphoma model EL4, 0.5 × 10^6^ cells were implanted into C57BL/6 J mice. To establish the colorectal model CT26, 2 × 10^6^ cells in 50% Matrigel (Corning) were implanted into the right flank of 7–10 week old female BALB/cAnNCrl mice. To establish the B cell lymphoma model A20, or the renal cancer model RENCA, 1 × 10^6^ cells were implanted into BALB/cAnNCrl mice. To establish the breast cancer model EMT6 and 4T1, 1 × 10^6^ or 0.5 × 10^6^ cells respectively were implanted subcutaneously into the right mammary fat pad of 7–10 week old female BALB/cAnNCrl mice. To establish the teratocarcinoma model F9, 2.0 × 10^6^ cells were implanted into 129S6/SvEvTac mice.

### Tumor collection for RNA-Seq and whole exome sequencing

When the calculated tumor volume was 400–500 mm^3^, mice were euthanized using slow fill CO_2_ euthanasia according to Pfizer approved methods. The tumors were collected using aseptic technique and the tumors were transferred to RNAse and DNAse free tubes (Thermo Scientific Catalog 374,320). Tumors were stored under liquid nitrogen until they were processed for RNA-Seq and WES.

### Whole exome sequencing

WES was conducted by Q^2^ solutions, USA using paired-end sequencing with read length of 2 × 100 bps. Raw reads were aligned to the UCSC mm10 reference genome using BWA (v 0.7.5) [34]. Picard and GATK tools were used for duplicates removal, reads realignment and recalibration. Variants were called using both Varscan 2 (v2.3.6) [35] and SomaticSniper (v1.0.4) [36]. Varscan 2 was performed using the *somatic* command with default parameters except --min-coverage was set to 20. The identified variants by varscan *somatic* were further filtered with *somaticFilter* and *processSomatic* commands using default parameters to obtain high confidence variant calls. Variant calls were made with SomaticSniper using default parameters and the results were further processed using scripts provided by the SomaticSniper package according to the suggestions from the manual to obtain high confidence variant calls. Variants from both Varscan 2 and SomaticSniper were annotated and filtered to obtain exonic variants using snpEFF (v4.1d) [20]. Further, variants potentially leading to altered protein functions were defined as those annotated with MODERATE or HIGH IMPACT by snpEFF. The intersection of variant calls from VarScan 2 and SomaticSniper predictions was used as the final variant call list. One hundred fifteen variants mapped to genes from TARGET database was further subjected to validation by Sanger sequencing at GeneWiz.

Mutational load in exomes was calculated based on the identified HIGH and MODERATE impact mutations in protein-coding genes (assuming 32 Mb [12] of protein-coding sequence). Median of Ts/Tv for each human tumor types was calculated based on data from Alexandrov et al. [14]. Median mutational load in exomes of human breast, lung squamous, lung adeno, colon, renal cell carcinomas and melanoma was calculated based on the identified nonsynonymous mutations in protein-coding genes (assuming 30 Mb [14] of protein-coding sequence) using data downloaded from cBioPortal (http://www.cbioportal.org/). To evaluate the mutation of human known cancer genes in syngeneic models, genes from the TARGET database were downloaded. Variants of cancer actionable genes were queried from OncoKB (http://oncokb.org/). Human mutation frequency data of TCGA samples for breast, lung squamous, colon, renal cell carcinomas and melanoma was downloaded from cBioPortal (http://www.cbioportal.org/). Subsequently, the mutation status of genes from TARGET database with at least 10% mutation frequency in TCGA samples was evaluated in syngeneic models that are of the same tissue origin.

### Neoantigen prediction

To predict neoantigens for each model, protein sequences for genes with predicted missense mutation were obtained from the Ensembl ftp site (ftp://ftp.ensembl.org/pub/release-84/fasta/mus_musculus/pep/). Two FASTA sequences were generated per variant site, wild type and mutant, with 10 amino acid sequences flanking each side of the variant site using pVAC-Seq [37]. The mouse haplotype (http://www.ebioscience.com/media/pdf/Mouse_Haplotype_Table.pdf) and candidate mutant epitopes for each variant were input to the IEDB MHC-I binding prediction tool. The IC50 for mutated epitopes with lengths of 8 to 11 amino acids was predicted using NetMHCpan (Vita et al. 2015), and peptides predicted to have IC50 values less than or equal to 500 nM and more favorable than the wild type peptide were identified. We evaluated the expression of the corresponding gene for each predicted epitope and required that the gene expression to be above 2 TPM. Comparison of mutational load and predicted neoantigens was performed using the spearman method in R.

### Transcription profiling (RNA-Seq)

RNA-Seq profiling was conducted by Q^2^ solutions, USA. RNA from 30 cell cultures and 21 tumor tissue samples corresponding to 10 syngeneic mouse models were pair-end sequenced with read length of 2 × 100 bps. Three replicates of cell culture and two replicates of tumor tissue were performed for each model. Raw reads were mapped to the UCSC mm10 reference genome using Bowtie 2 (v2.2.5) [38]. Expected counts and normalized expression levels of genes in transcripts per million (TPM) were generated by RSEM (v1.2.20) [39]. Genes specifically up-regulated in CT26 in vivo tumor samples compared to CT26 grown in in vitro cell culture and in vivo tumor samples from other models were obtained as follows. First, genes significantly up-regulated in CT26 in vivo tumor samples compared to CT26 in vitro samples were identified using the DESeq2 package with criteria of adjusted *p*-value <= 0.01 and fold change > = 2. Secondly, genes up-regulated in CT26 in vivo tumors compared to in vivo tumors from other models were obtained using the DESeq2 package with criteria of adjusted *p*-value <= 0.01 and fold change > = 2. The final gene list was obtained from intersecting the above two gene lists and then was subjected to pathway analysis. All pathway enrichment analysis was performed using Ingenuity Pathway Analysis (IPA, Ingenuity® Pathway Analysis (IPA®)). To compare gene expression of markers of immune cell type, immune cell activation and immune suppression between cells grown in vitro and tumor tissues from the transplantation, standardized log2 (TPM) values were plotted in a heat map (Partek® Genomics Suite®). Unsupervised hierarchical clustering analysis of gene expression of in vivo tumor samples was performed using the Partek® Genomics Suite® (Euclidean distance, average linkage clustering method). Cytolytic activity (CYT) was defined as the log-average (geometric mean) of Gzma and Prf1 expression value (TPM) as described by Rooney et al. [17]. Human cytolytic activity data were downloaded from Rooney et al. [17]. To compare ratio of the E-cadherin and vimentin gene expression between syngeneic models and human tumors, RNA-Seq data from TCGA were used. The ratio was calculated with the expression value (TPM) of E-cadherin and vimentin.

### In silico immune cell deconvolution

In silico immune cell deconvolution of in vivo tumor samples, either in vitro or in vivo, was performed on RNA-Seq profiling data using a nuSVR approach for mouse samples that is similar to approaches recently developed for human samples [21]. To establish a mouse immune cell-specific gene signature matrix, we downloaded RNA-Seq profiling data from 11 purified mouse immune cell subsets generated by the Immunological Genome Project (https://www.immgen.org/). These 11 immune cell subsets span all major hematopoietic lineages, and were double-sorted by flow cytometry from the spleen or peritoneal cavity of a 5-week old male C57BL/6 J mouse (Jackson Laboratory). A list of the 11 immune cell subsets and the sorting markers are in Additional file 5: Table S4. More details about the RNA-Seq dataset can be found in the Sequence Read Archive (https://www.ncbi.nlm.nih.gov/sra) under accession PRJNA281360.

Raw RNA-Seq reads were aligned to the mouse reference transcriptome/genome (mm10) using Bowtie 2 [38] and summarized into gene-level transcripts per million (TPM) measures by RSEM [39]. TPM values were further quantile normalized before subsequent analysis. We extended a procedure for the selection and optimization of an immune cell specific gene signature matrix to mouse samples that is similar to approaches recently developed for human samples [21]. Since there are no biological replicates per immune cell type, we used a Z-statistic to test whether any gene is significantly over-expressed in one immune cell subset versus all others. We kept all candidate genes for each cell type that have a q-value from the Z-test less than 0.01 and are expressed at least two-fold above the 3rd quartile expression values of all genes in a cell type. An equal number of candidate genes from each cell type (sorted by expression fold change from the cell type of interest and the mean from all other cell types) were combined to form a gene signature matrix, where the optimal number of candidate genes was determined using a conditional number minimization procedure [21]. This process selected a 577-gene signature matrix for the 11 mouse immune cell subsets.

With this immune cell-specific gene signature matrix, we performed deconvolution of bulk tumor profiles using a nuSVR algorithm [40]. Related methods for deconvolution of immune subset have been established for human samples [21]. Our approach incorporates unique fingerprints derived for mice immune components. A deconvolution *p*-value was calculated for each sample which indicates whether there is significant presence of any immune cells (among the 11 immune cell subsets included in the gene signature matrix). A *p*-value cutoff of 0.1 was used to indicate significant deconvolution. In vitro tumor samples were used as negative controls for deconvolution as they should not have any immune cells, except for the EL4 and A20 hematological tumor models. Output from a significant deconvolution is relative fractions of the 11 immune cell subsets in the bulk tumor samples. The fraction of each immune cell subsets is relative to the total leukocyte content (e.g. CD45+ cells) in the sample and should sum up to 100%. Stacked bar charts were used to display these immune cell subset fractions as the average of biological replicates of a tumor model.

Total T-cell fraction is calculated as the sum of all predicted T-cell subsets (mouse: CD4+, CD8+, Treg, and gamma-delta T-cells; human: CD8+, CD4+ naïve, CD4+ memory RO unactivated, CD4+ memory RO activated, T cells follicular helper, T cells gamma delta, Tregs). Human leukocyte infiltration data are downloaded from Gentles et al. [22].

### Histology and immunohistochemistry

Five micron sections were cut onto charged slides, dried, deparaffinized in xylene and rehydrated with graded alcohols to distilled H_2_O. For Hematoxylin and Eosin staining, sections were submerged in Tacha’s Auto Hematoxylin (Biocare Medical, Concord, CA, USA) for 1 min then rinsed in distilled H_2_O until clear. Slides were then submerged in tap water with agitation for 1 min followed by 1 min in 80% Reagent Alcohol (Thermo Fisher, Histoprep). Sections were then submerged in Eosin Y (Thermo Fisher) for 1.5 min followed by three 5 s washes in 95% Reagent Alcohol (Thermo Fisher, Histoprep), two 5 s washes in 100% Reagent Alcohol (Thermo Fisher, Histoprep), and finally in Xylene (Thermo Fisher, Histoprep) before being coverslipped with Permount mounting medium (Fisher Scientific Co. L.L.C., Pittsburgh, PA, USA). Immunohistochemistry heat-induced epitope retrieval was performed in the Retriever 2000 pressure cooker (Electron Microscopy Sciences, Hatfield, PA, USA) in Citrate Buffer pH 6.0 (Invitrogen, Carlsbad, CA, USA) or Borg buffer pH 9.5 (Biocare Medical, Concord, CA, USA) and cooled to room temperature for 20 min. Endogenous peroxidase activity was inactivated with Peroxidazed 1 (Biocare Medical, Concord, CA, USA) for 10 min. Non-specific protein interactions were blocked for 10 min with Background Punisher (Biocare Medical, Concord, CA, USA). Sections were incubated with primary antibodies, CD11b (AbCam, EPR1334, Citrate, 0.088 μg/ml), F4/80 (Spring Bioscience, M4152, Citrate 2.5 μg/ml), RA3-6B2, Borg, 2.5 μg/ml), CD3 (Spring Bioscience, SP162, Borg, 1.33 μg/ml), vimentin (Cell Marque, SP20, Citrate, 3 μg/ml), E-cadherin (AbCam, EP700Y, Citrate, 0.18 μg/ml), for 1 h, washed in TBS and incubated with SignalStain Boost IHC Detection Reagent (Cell Signaling Technologies, Beverly, MA, USA) for 30 min. Following washes in TBS, immunoreactivity was visualized by development with 3,3′-diaminobenzidine (DAB+, Dako, Carpinteria, CA, USA) for 5 min. Immunostained sections were briefly counterstained with CAT Hematoxylin, washed in tap water, dehydrated in graded alcohols, cleared in xylene, and coverslipped with Permount mounting medium (Fisher Scientific Co. L.L.C., Pittsburgh, PA, USA).

### Proteomics acquisition and data analysis

All tissues were dissociated in 100 mM sodium carbonate using TissueLyzer II (QIAGEN) for 0.5 min at a rate of 0.30 repetitions three times and stored on ice for 30 s between the dissociation cycles. The cells grown in vitro were detached with CellStripper (Mediatech), washed with cold PBS twice and cell pellets were collected. 100 mM sodium carbonate containing 1 mM DTT, pH 11.5 was used to lyse cells by incubating the cell suspension on ice for 60 min. For all tissue and cell protein lysates the pH was adjusted to pH 8 by the addition of Tris-HCl buffer at pH 7.0 and a final concentration of 2 mM MgCl_2_. Universal Nuclease (ThermoFisher) was used to dechromatinize nuclear DNA for 30 min on ice. The membrane fraction was isolated by centrifugation at 20,000 g for 60 min at 4 °C and the supernatant was transferred to a fresh tube as the soluble fraction. The precipitated fraction following this centrifugation is membrane-protein enriched and is referred to as the “membrane” fraction, whereas supernatant is referred to as the “soluble” proteome fractions. The resulting membrane fraction (pellet) was solubilized using RIPA buffer (25 mM TrisHCl, pH 7.6, 150 mM NaCl, 1% SDS, 1% sodium deoxycholate, 1% NP-40 and 1X protease inhibitor) to solubilize membrane proteins, whereas the soluble fraction was used directly for subsequent processing following determination of protein concentration by BCA assay. Each fraction was processed separately using filter-assisted sample proteolysis (FASP). Briefly, 50 μg of protein was loaded on to a PES 100 KDa filter, washed 4 times with 8 M Urea in 100 mM Tris-HCl buffer, pH 8.5. Proteins were reduced with 5 mM DTT (freshly prepared) and alkylated with 10 mM idoacetamide in the dark. Samples were then washed three times with freshly prepared 25 mM ammonium bicarbonate buffer and digested with trypsin/LysC with protein: enzyme at a 25:1 weight ratio overnight. Following digestion, the peptides were captured by centrifugation at 15,000 g for 20 min, followed by adjusting the solution pH to acidic by adding 10% formic acid to the final concentration of 0.25% formic acid.

Each sample was acquired on a Thermo Scientific™ Q Exactive™ Hybrid Quadrupole-Orbitrap Mass Spectrometer fitted with a Dionex nano liquid chromatography and EASY-Spray™ Ion source. The tryptic digest was loaded onto a reversed-phase pre-column (C18 trap column, Acclaim PepMap 100, 100 μm × 2 cm, Thermo Fisher Scientific, Inc.). Peptide separation was conducted via nano-LC using a 75 μm × 150 mm PepMap C18 EASY-Spray column (3 μm, 100 Å particles, Thermo Scientific, Inc.). The gradient was comprised of an increase from 2 to 35% mobile phase B (0.1% formic acid in acetonitrile) over 160 min, followed by 35 to 80% B over 10 min and a hold at 80% B for the last 10 min, all at a fixed flow rate of 300 nl/min in an UltiMat 3000 RSLCnano system (Thermo Fisher Scientific, Inc.). Q Exactive runs were operated with data dependent top 10 method and the parameters were as follows: resolution 70,000 at m/z 200 for MS1 with a scan range of 300–1650 m/z, a predictive AGC target of 3 × 10^6^ and a maximum injection time of 100 ms; 17,500 at m/z 200 for dd-MS2 with a predictive AGC target of 2 × 10^5^, maximum injection time 120 ms, NCE of 25, 20s dynamic exclusion and underfill ratio 3%.

For soluble and membrane fractions, peptides were identified and quantified for label-free protein quantification from raw mass spectrometric files using MaxQuant software (version: 1.6.1.0) [41], respectively. Database searching was performed in MaxQuant using the Andromeda search engine [42] against the mouse Uniprot database (57,208 entries, May 2017 version) supplemented with our internal non-redundant virus protein sequences containing 903 ENA and 260 GenBank entries. Andromeda search parameters for protein identification were set as follows: maximum mass tolerance of 20 ppm for the first search, 4.5 ppm for the precursor ions after non-linear recalibration and 20 ppm for fragment ions; digestion was set to specific “Trypsin/P” allowing max missed cleavage events of two. Oxidation of methionine and protein N-terminal acetylation were set as variable modifications. Carboxyamidomethylation of cysteines was specified as a fixed modification. A total of three modifications were allowed per peptide. Minimal required peptide length was seven amino acids. MaxQuant LFQ “match between run” option was enabled with a match time window of 0.7 min after retention time alignment. “Requires MS/MS for label-free quantification (LFQ) comparisons” was not enabled to allow maximum MS peak features. Confidently identified proteins were required to have a minimum of two matched peptides with one peptide being uniquely matched at a false discovery rate (FDR) of less than 1%. Proteins were quantified by delayed normalization computed in MaxQuant’s label-free quantification option [43].

LFQ intensity data by protein group was further analyzed in Perseus (version: 1.6.0.7) [44]. All proteins “identified by site”, “reverse” and contaminants were removed. LFQ intensity value was log2 transformed and the resulting data matrix was further filtered requiring minimal valid values of 100% in at least one model (Additional file 6: Table S5).

Proteins that are significantly over-expressed in CT26 in vivo tumor samples were obtained by pairwise comparison of the log2 transformed LFQ value of CT26 in vivo tumor samples from membrane fraction or soluble fraction to one of the other tumor models by applying a filter requiring a valid value of > = 75% in at least one pair and using student t-test within Persues. Proteins with FDR (Benjamini-Hochberg) < = 0.05 and fold change > = 2 in all comparisons as well as CT26 in vivo vs. CT26 in vitro samples were selected for further pathway and function enrichment analysis. If proteins were detected in both membrane and soluble fractions, the identification from the fraction with the higher LFQ values of the proteins will be kept. Pathway, function enrichment and network analysis were performed using Ingenuity Pathway Analysis (IPA, Ingenuity® Pathway Analysis (IPA®).

Hierarchical clustering of virus protein was performed within R using the Euclidean distance and the complete linkage clustering method. LFQ values of identified virus proteins were first log2 transformed and then scaled separately within in vitro samples and in vivo samples (which included both tumors and normal tails) before clustering analysis.

### Antibodies to mouse immune checkpoint proteins

Rat IgG2a to mouse PD-1/CD279 (clone RMP 1–14) was purchased from BioXcell and was dosed in vivo at 10 mpk i.v. q3dx3. Hamster IgG to mouse CTLA4/CD152 (clone 9H10) was purchased from BioXcell and was dosed in vivo at 10 mpk i.v. q3dx3.

### In vivo evaluation of antibodies to mouse immune checkpoint proteins

When the average tumor volume reached approximately 70 to 160 mm^3^, mice were randomized into treatment groups, with 10 mice in each treatment group. Antibodies or vehicle (PBS) were administered intravenously on study day 0 and then the animals were dosed once every 3 days for 3 doses. Tumors were measured 1–3 times per week and tumor volume was calculated as volume (mm^3^) = (width x width x length)/2.

## Supplementary information

**Additional file 1: Table S1.** Variant calls by WES.

**Additional file 2: Figure S1.** Mutation landscape of syngeneic models and types of somatic variants for each model. **Figure S2.** A. Ratio of Ts (Transition) to Tv (Transversion) substitution mutation in each syngeneic model; dashed lines represent maximum, median and minimum of median Ts/Tv from each human tumor types (data from (Alexandrov et al. 2013)) respectively. B. Single nucleotide mutation changes in each syngeneic model. **Figure S3.** E-cadherin and vimentin stain in syngeneic models. **Figure S4.** A. Correlation of neoantigen with mutational load in syngeneic models. B. Correlation of neoantigen with cytolytic activity in syngeneic models. The correlation was calculated using spearman method in R. numMutation: number of missense mutation. **Figure S5.** Response of syngeneic tumor models to anti-CTLA4, or anti PD-1. All mice were dosed intravenously. Individual tumor volumes are shown for 10 mice treated with PBS (black) or 10 mg/kg anti-CTLA4 (9H10) (red trace), or 10 mg/kg anti-PD-1 (RMP 1–14) (blue trace). All 10 mice bearing CT26 tumors dosed with anti-CTLA4 had no measurable tumor from study day 25 until measurements ended on study day 238. Comparison of mean tumor volumes was analyzed using log-transformed ANOVA. *P*-values are shown if there was a statistical difference between vehicle and antibody-treated groups. n.s. not significant. **Figure S6.** A. Expression of Gzma and Prf1 in syngeneic model grown in vitro (Cell) and in vivo (Tissue) across models based on protein expression. B. Cytolytic activity of syngeneic models. Cytolytic activity (CYT) is defined as the log-average (geometric mean) of Gzma and Prf1 protein expression. C. Protein expression of Gzma in soluble fraction. LFQ: label free quantitation. **Figure S7.** Gene expression of β-catenin, β-catenin target genes (gene list from (Spranger et al. 2015)) and epigenetic modulators (Ezh2, Dnmt1) across syngeneic models. **Figure S8.** CTLA4 expression in syngeneic in vivo tumor samples.

**Additional file 3: Table S2.** Validation of 115 variant calls by Sanger sequencing.

**Additional file 4: Table S3.** Gene expression data by RNA-Seq.

**Additional file 5: Table S4.**  A list of the 11 immune cell subsets and sorting markers.

**Additional file 6: Table S5.** Protein expression data by proteomics.

## Data Availability

RNA-Seq data is available from SRA with accession number PRJNA505989 (https://www.ncbi.nlm.nih.gov/bioproject/PRJNA505989). WES data is available from SRA with accession number PRJNA506146 (https://www.ncbi.nlm.nih.gov/bioproject/PRJNA506146). The mass spectrometry proteomics data have been deposited to the ProteomeXchange Consortium via the PRIDE [45] partner repository with the dataset identifier PXD011885 (https://www.ebi.ac.uk/pride/archive/projects/PXD015037).

## Abbreviations

BRCA: Breast invasive carcinoma
CDXs: Cell-line derived xenografts
COAD: Colon adenocarcinoma
CYT: Cytolytic activity
FASP: Filter-assisted sample proteolysis
FDR: False discovery rate
GEMMs: Genetically engineered mouse models
IACUC: Institutional Animal Care and Use Committee
IHC: Immunohistochemistry
KIRC: Kidney renal clear cell carcinoma
LFQ: Label-free quantification
LLCsr: Lewis lung carcinoma
LUAD: Lung adenocarcinoma
LUSC: Lung squamous cell carcinoma
nuSVR: nu-support vector regression
PDXs: Patient-derived xenografts
PRC2: Polycomb repressive complex 2
SKCM: Skin cutaneous Melanoma
SNVs: Single nucleotide variants
TARGET: Tumor Alterations Relevant for Genomics-driven Therapy
TPM: Transcripts per million
Ts: Transition
Tv: Transversion
WES: Whole exome sequencing
