# Supplementary material for: Comparison of the molecular and cellular phenotypes of common mouse syngeneic models with human tumors
Source: BMC Genomics. 2020 Jan 2;21:2. doi: 10.1186/s12864-019-6344-3 (PMC6941261; doi:10.1186/s12864-019-6344-3)
Supplement: Supplementary file 2 — Additional file 2: Figure S1. Mutation landscape of syngeneic models and types of somatic variants for each model. Figure S2. A. Ratio of Ts (Transition) to Tv (Transversion) substitution mutation in each syngeneic model; dashed lines represent maximum, median and minimum of median Ts/Tv from each human tumor types (data from (Alexandrov et al. 2013)) respectively. B. Single nucleotide mutation changes in each syngeneic model. Figure S3. E-cadherin and vimentin stain in syngeneic models. Figure S4. A. Correlation of neoantigen with mutational load in syngeneic models. B. Correlation of neoantigen with cytolytic activity in syngeneic models. The correlation was calculated using spearman method in R. numMutation: number of missense mutation. Figure S5. Response of syngeneic tumor models to anti-CTLA4, or anti PD-1. All mice were dosed intravenously. Individual tumor volumes are shown for 10 mice treated with PBS (black) or 10 mg/kg anti-CTLA4 (9H10) (red trace), or 10 mg/kg anti-PD-1 (RMP 1–14) (blue trace). All 10 mice bearing CT26 tumors dosed with anti-CTLA4 had no measurable tumor from study day 25 until measurements ended on study day 238. Comparison of mean tumor volumes was analyzed using log-transformed ANOVA. P-values are shown if there was a statistical difference between vehicle and antibody-treated groups. n.s. not significant. Figure S6. A. Expression of Gzma and Prf1 in syngeneic model grown in vitro (Cell) and in vivo (Tissue) across models based on protein expression. B. Cytolytic activity of syngeneic models. Cytolytic activity (CYT) is defined as the log-average (geometric mean) of Gzma and Prf1 protein expression. C. Protein expression of Gzma in soluble fraction. LFQ: label free quantitation. Figure S7. Gene expression of β-catenin, β-catenin target genes (gene list from (Spranger et al. 2015)) and epigenetic modulators (Ezh2, Dnmt1) across syngeneic models. Figure S8. CTLA4 expression in syngeneic in vivo tumor samples. [file 12864_2019_6344_MOESM2_ESM.pdf]

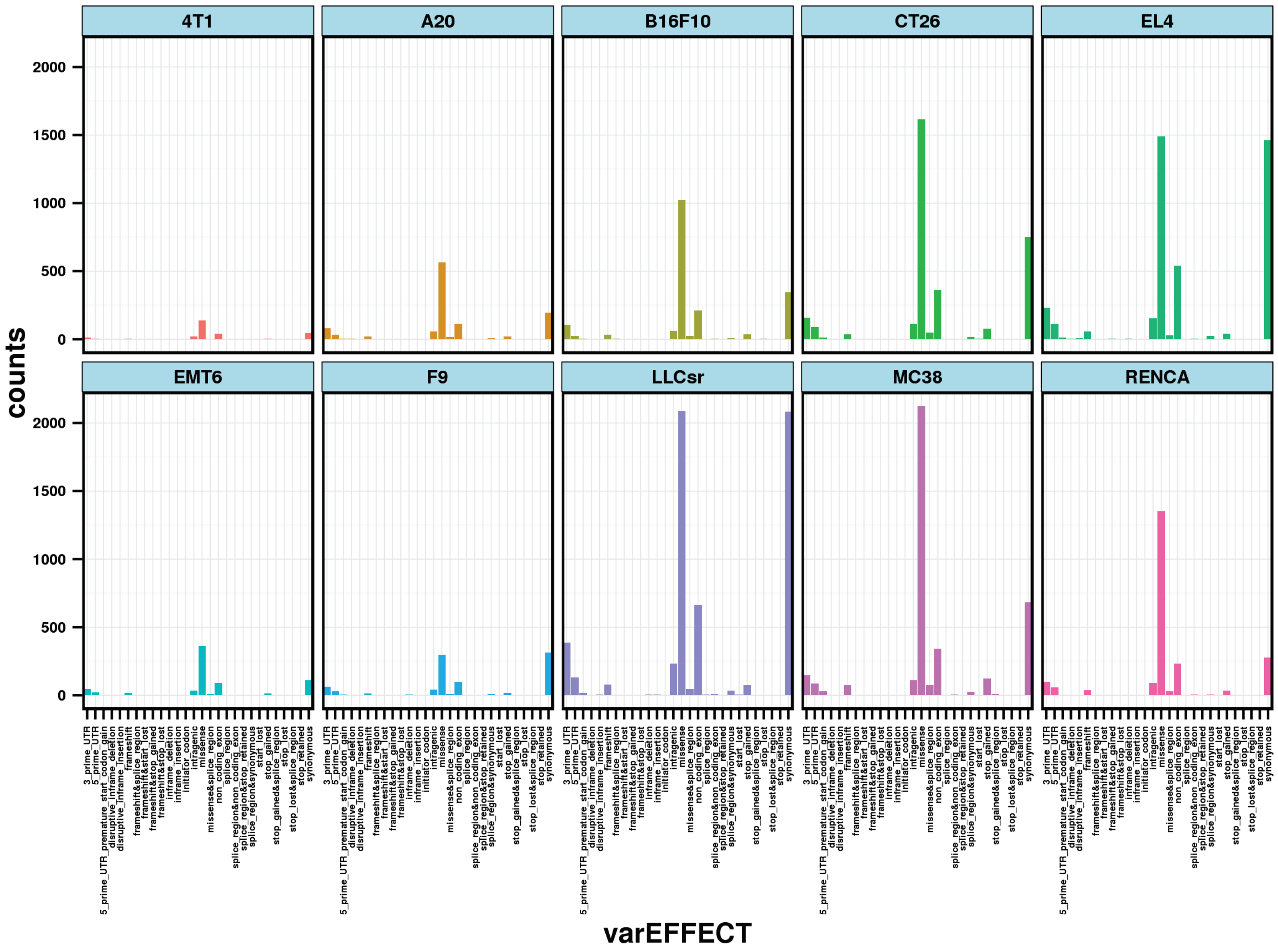

Figure S1. Mutation landscape of syngeneic models and types of somatic variants for each model.

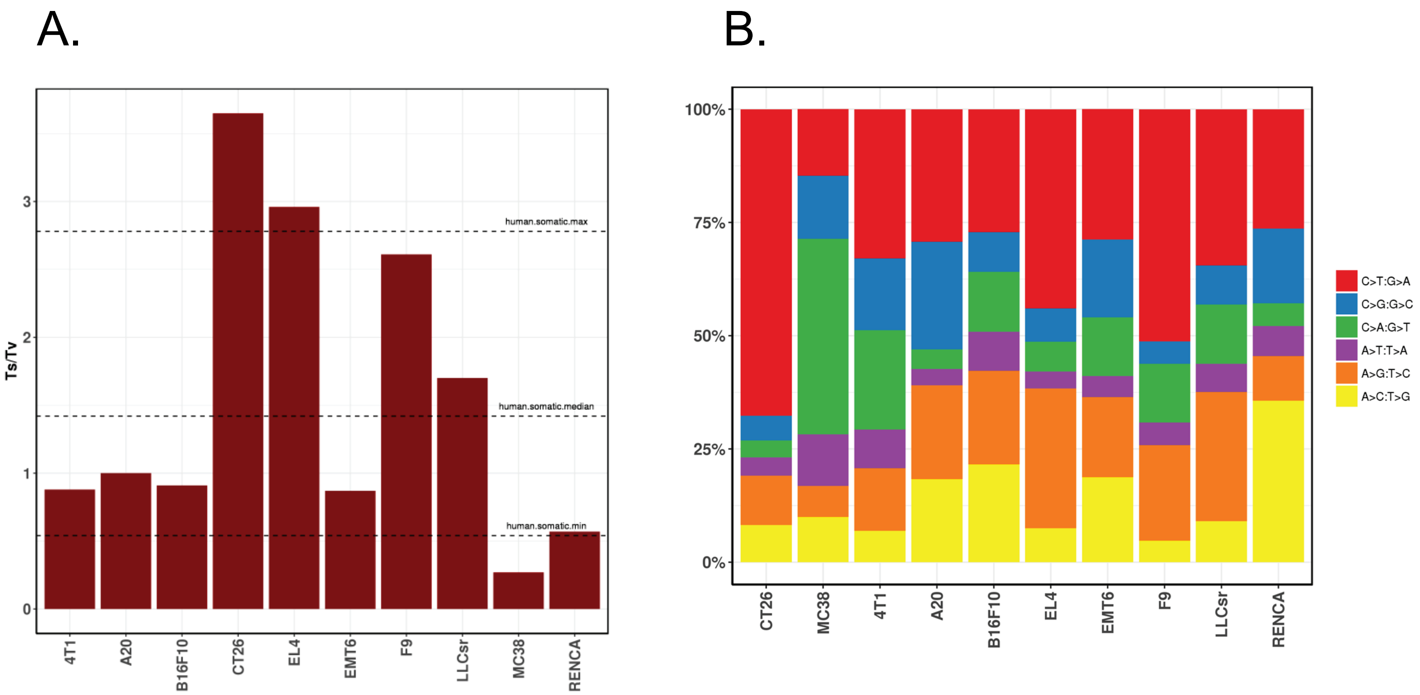

Figure S2. A. Ratio of Ts (Transition) to Tv (Transversion) substitution mutation in each syngeneic model; dashed lines represent maximum, median and minimum of median Ts/Tv from each human tumor types (data from (Alexandrov et al. 2013)) respectively. B. Single nucleotide mutation changes in each syngeneic model.

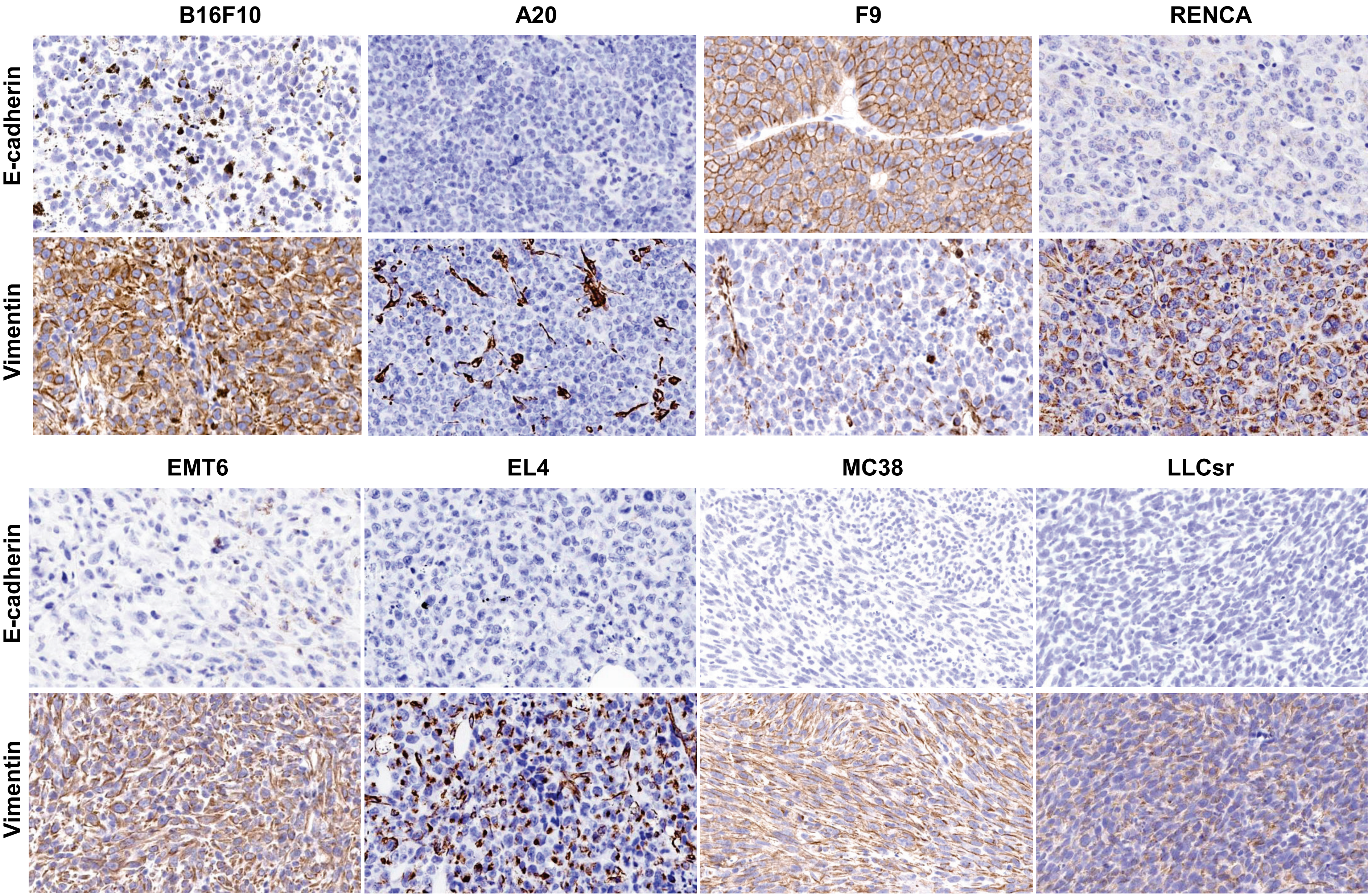

Figure S3. E-cadherin and vimentin stain in syngeneic models.

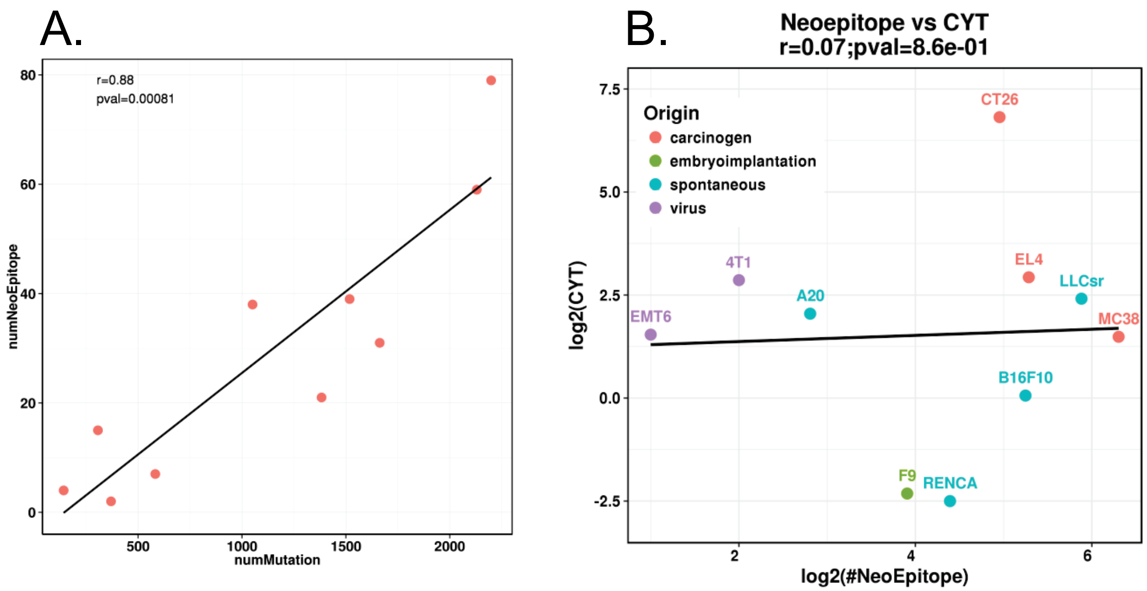

Figure S4. A. Correlation of neoantigen with mutational load in syngeneic models.
B. Correlation of neoantigen with cytolytic activity in syngeneic models.
The correlation was calculated using spearman method in R.
numMutation: number of missense mutation.

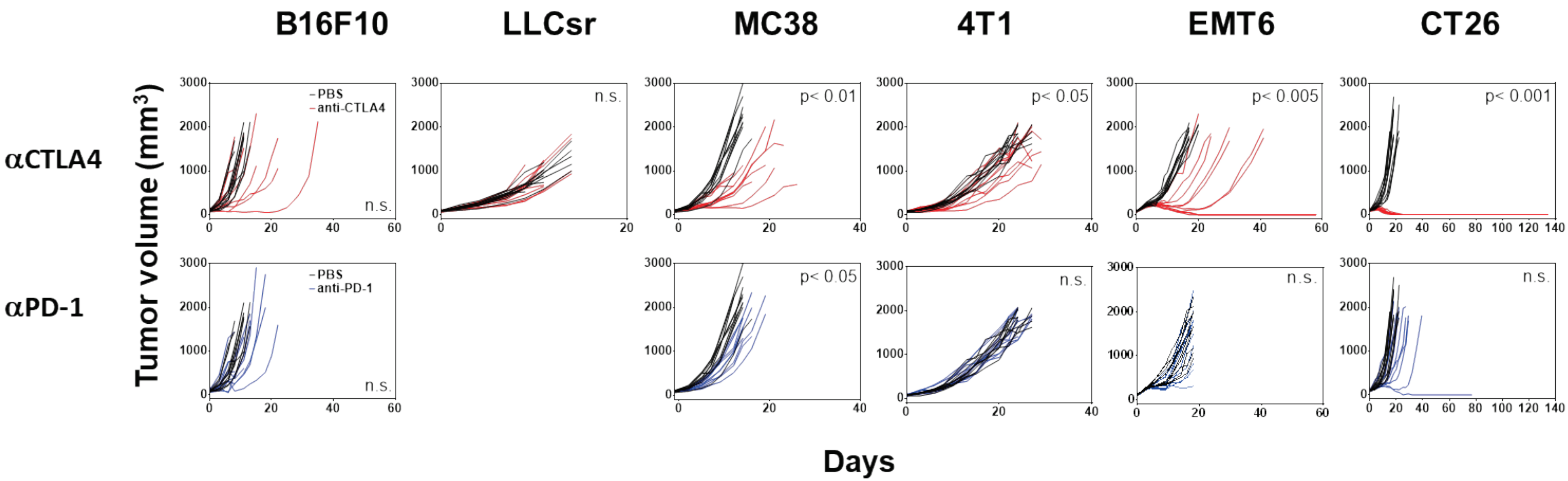

Figure S5. Response of syngeneic tumor models to anti-CTLA4, or anti PD-1. All mice were dosed intravenously. Individual tumor volumes are shown for 10 mice treated with PBS (black) or 10 mg/kg anti-CTLA4 (9H10) (red trace), or 10 mg/kg anti-PD-1 (RMP 1-14) (blue trace). All 10 mice bearing CT26 tumors dosed with anti-CTLA4 had no measurable tumor from study day 25 until measurements ended on study day 238. Comparison of mean tumor volumes was analyzed using log-transformed ANOVA. P-values are shown if there was a statistical difference between vehicle and antibody-treated groups. n.s. not significant.

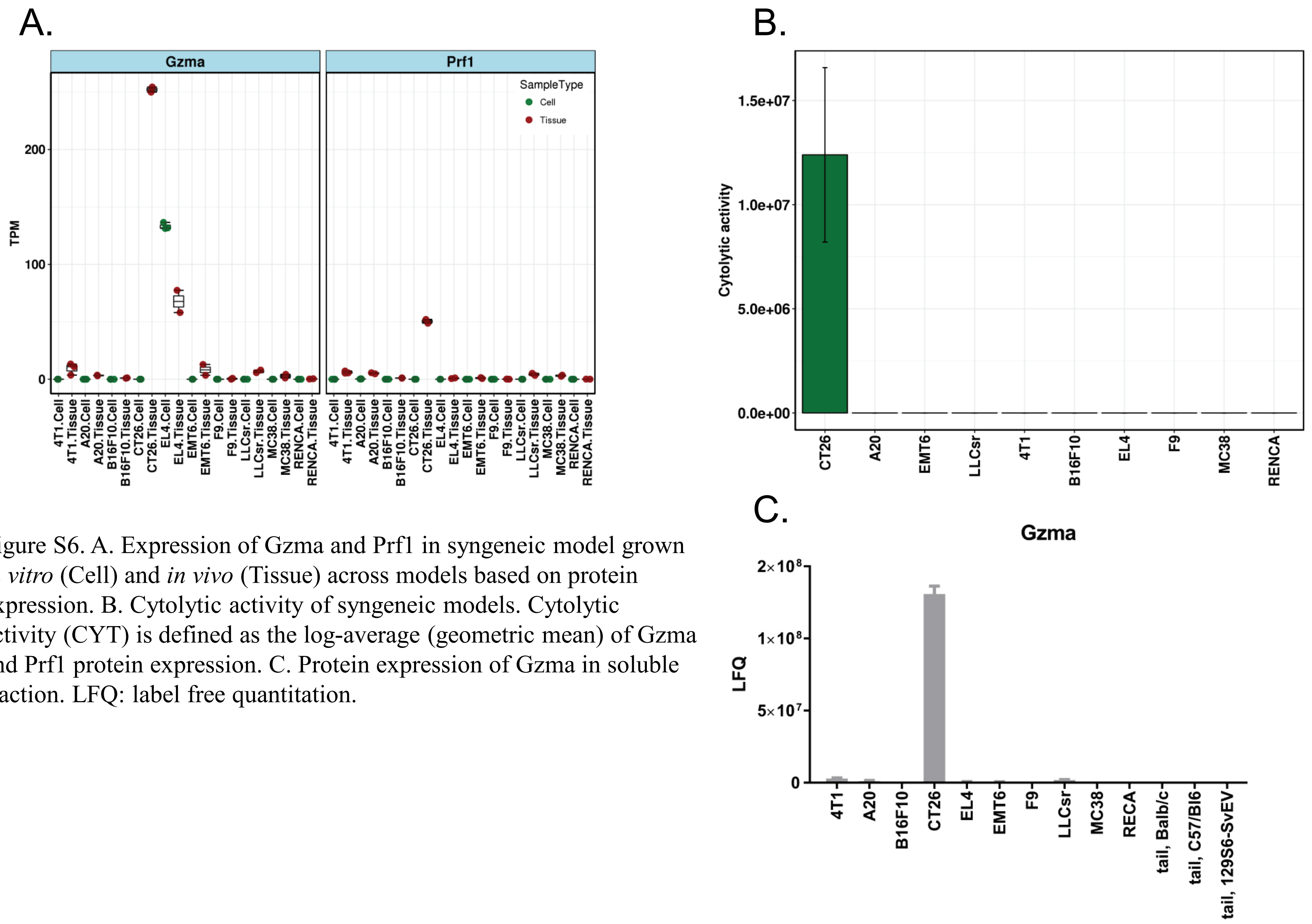

Figure S6. A. Expression of Gzma and Prf1 in syngeneic model grown *in vitro* (Cell) and *in vivo* (Tissue) across models based on protein expression. B. Cytolytic activity of syngeneic models. Cytolytic activity (CYT) is defined as the log-average (geometric mean) of Gzma and Prf1 protein expression. C. Protein expression of Gzma in soluble fraction. LFQ: label free quantitation.

β-catenin

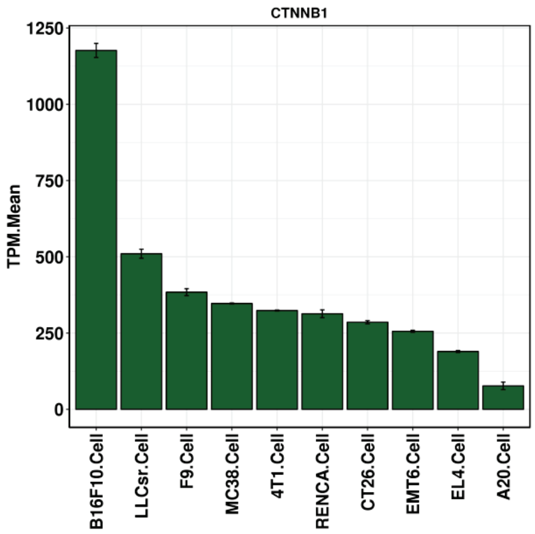

β-catenin target genes

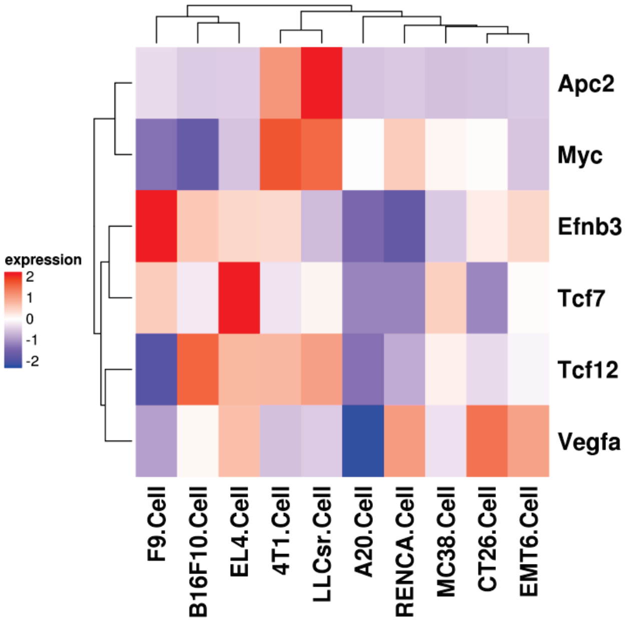

Epigenetic modulators

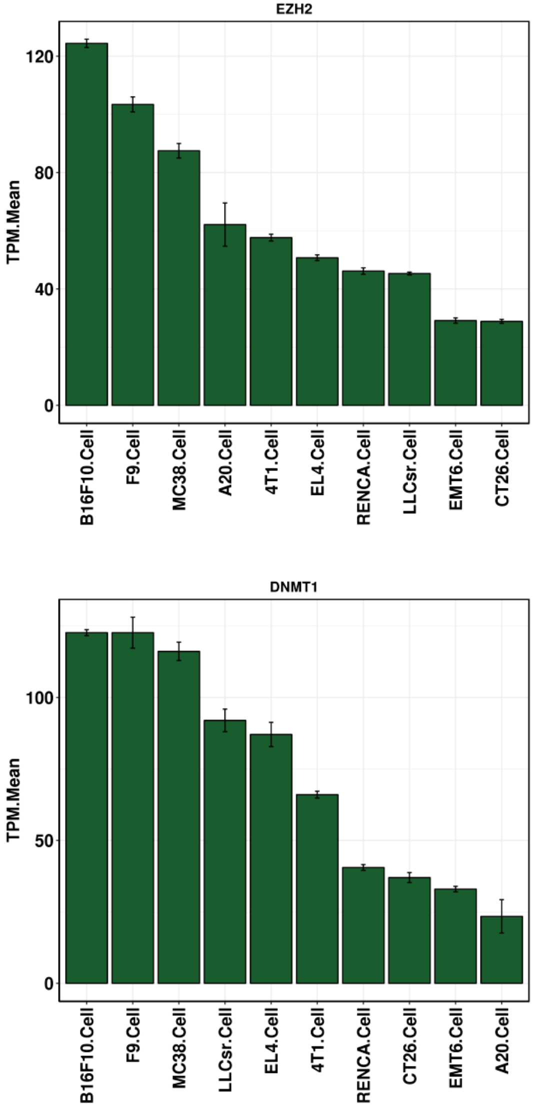

Figure S7. Gene expression of β-catenin, β-catenin target genes (gene list from (Spranger et al. 2015)) and epigenetic modulators (Ezh2, Dnmt1) across syngeneic models.

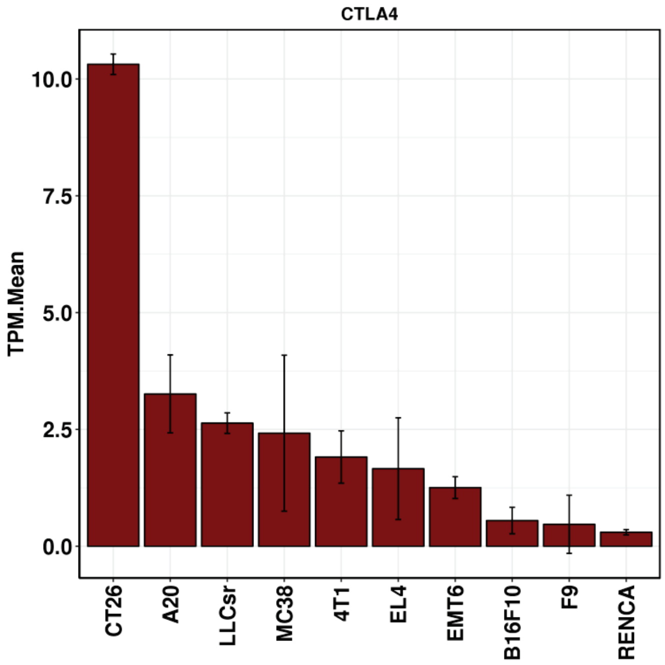

Figure S8. CTLA4 expression in syngeneic *in vivo* tumor samples.
